# Point-of-care Ultrasound for Suspected Pectoralis Major Rupture: A Case Report

**DOI:** 10.5811/cpcem.2020.10.50802

**Published:** 2021-01-27

**Authors:** Nathanael Franks, Jeremiah Gress, Ryan Joseph

**Affiliations:** * Long School of Medicine, UT Health San Antonio, San Antonio, Texas; † UT Health San Antonio, Department of Emergency Medicine, San Antonio, Texas

**Keywords:** Point-of-care ultrasound, POCUS, pectoralis major injury, pectoralis major rupture, case report

## Abstract

**Introduction:**

Pectoralis major muscle injuries are relatively uncommon and occur secondary to weightlifting in nearly 50% of cases. Tendon tears occur almost exclusively in males between 20–40 years old and are heavily associated with anabolic androgenic steroid use. While magnetic resonance imaging is often considered the modality of choice, its availability is often limited in the emergency department (ED). In contrast, point-of-care ultrasound (POCUS) is commonly available in the ED and can be used to help confirm the diagnosis and hasten disposition.

**Case Report:**

We report a case of a 28-year-old male competitive weightlifter with a history of chronic anabolic steroid use who presented to the ED with acute left shoulder pain after weightlifting. History and physical exam were concerning for pectoralis major rupture, and POCUS confirmed the diagnosis.

**Conclusion:**

Prompt evaluation and radiographic confirmation is key in ensuring good patient outcomes in pectoralis major tears. Therefore, proficiency of emergency physicians in musculoskeletal POCUS as an adjunct to estimate the extent of injury is important for expediting disposition and and promptly involving orthopedic surgery evaluation.

## INTRODUCTION

Injuries of the pectoralis major muscle are relatively uncommon, and roughly 50% have been reported after weightlifting.^1^–^3^ Bench press is a common culprit due to the excessive tension put on an eccentrically contracted muscle.^1^–^3^ Less commonly, injuries occur after direct trauma causing forced abduction and external rotation of the upper extremity.^4^ Tendon tears occur almost exclusively in males between 20–40 years old and are heavily associated with anabolic androgenic steroid use.^5^,^6^ Other risk factors for major tendon rupture in general include Black race, young age, male gender, and sports participation.^7^ While magnetic resonance imaging (MRI) is often considered the modality of choice for the evaluation of pectoralis major tears due to its ability to differentiate the site, grade, and chronicity, it is expensive, time-consuming, and often unavailable in the emergency department (ED) setting.^8^,^9^ However, point-of-care ultrasound (POCUS) can be used in the ED due to its low cost and rapid availability to guide evaluation and treatment for patients.^4^

**Image 1:**
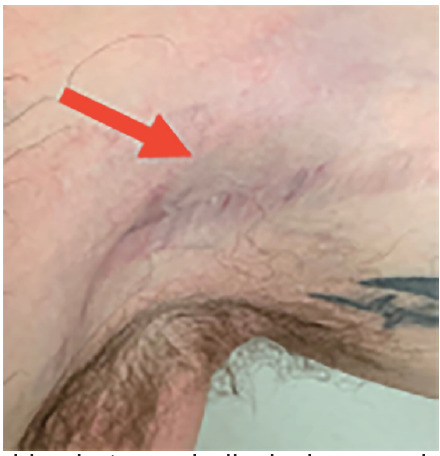
Bedside photograph displaying prominent ecchymoses (red arrow) over patient’s left axilla.

**Image 2:**
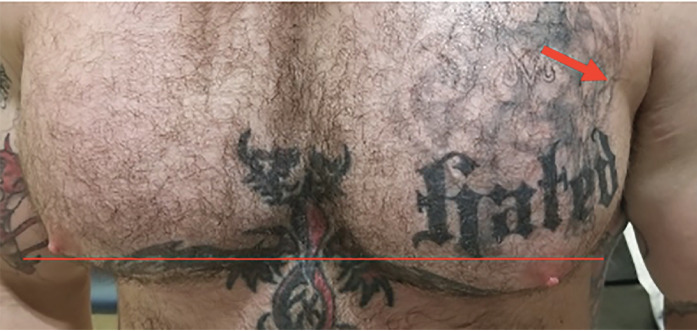
Photograph displaying asymmetry of patient’s nipple height (horizonal red line) following injury and subtle indentation (red arrow) over the lateral left pectoralis.

**Image 3:**
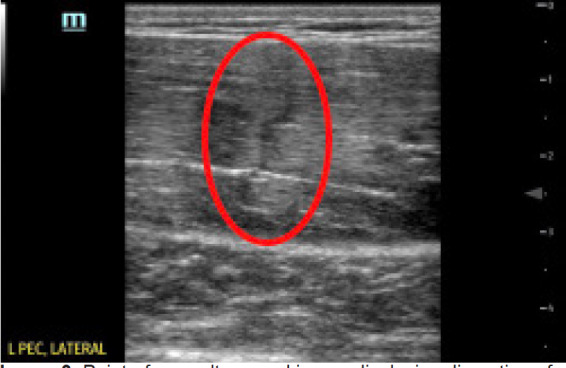
Point-of-care ultrasound image displaying disruption of the distal pectoralis major muscle bellies with vertical interruption noted (red circle).

**Image 4:**
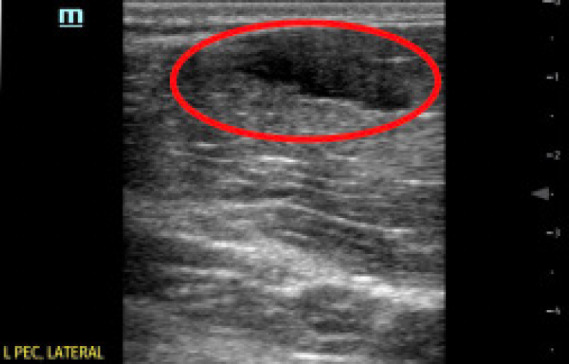
Point-of-care ultrasound image displaying hypoechoic hematoma formation (red circle) in the distal portion of pectoralis major muscle bellies.

A 28-year-old male competitive weightlifter with a history of chronic anabolic androgenic steroid use presented to the ED with left shoulder pain. The patient reported weightlifting several hours prior, and while performing dumbbell flys with 140 pounds in each arm he felt and heard a sudden “pop” with immediate loss of strength in his left shoulder. The patient endorsed constant pain at the site since the injury, which was exacerbated by arm movement. He denied any numbness or tingling. On physical exam of the left upper extremity, the skin was intact with significant bruising in the axilla (Image 1). There was asymmetric loss of normal contour of the left pectoralis major, and the left nipple was noted to be lower than the right (Image 2). Tenderness to palpation was reported across the left pectoral region, left axilla, and left anterior deltoid. Additionally, there was decreased strength with arm adduction and increased pain with internal rotation at the shoulder. Given the convincing clinical history and physical exam for pectoralis major rupture, POCUS was used for further evaluation. On sonography there were hypoechoic disruptions of muscular striations, hematoma formation, and pectoralis major muscle retraction noted at the site of injury, helping to confirm the pectoralis major rupture (Images 3 and 4). Radiographs of the left shoulder were performed showing no osseous injury. The orthopedic surgery team was consulted and scheduled the patient for magnetic resonance imaging (MRI) and prompt outpatient follow-up with a shoulder subspecialist.

**CPC-EM Capsule**

What do we already know about this clinical entity?

Pectoralis major tears are relatively uncommon and heavily associated with anabolic steroid use. Magnetic resonance imaging, the gold standard for diagnosis, is time consuming, expensive, and often unavailable for such applications in the emergency department.

What makes this presentation of disease reportable?

The confirmation of the diagnosis with point-of-care ultrasound in the emergency department makes this a unique case.

What is the major learning point?

Point-of-care ultrasound can help to quickly confirm the diagnosis of pectoralis major tears and make appropriate consultations for follow-up and further management.

How might this improve emergency medicine practice?

By making or confirming the diagnosis of a pectoralis major tear faster, we can get patients appropriate follow-up or specialty consultation more efficiently and avoid other, more expensive imaging modalities.

## DISCUSSION

The pectoralis major is a complex, fan-shaped muscle comprised of a clavicular head, originating from the medial half of the clavicle, and a sternocostal head, originating from the anterior sternum, costal cartilages of ribs 1–7, and the aponeurosis of the external oblique.^10^ The two heads coalesce into a common tendon and insert into the lateral lip of the intertubercular sulcus of the humerus.^10^ Rupture occurs most commonly in patients with a history of weightlifting, causing disruption of the distal humeral enthesis.^1^–^3^ Pectoralis major rupture in this patient was strongly suggested by the clinical presentation, but the availability of POCUS to confirm our suspicion assisted in expediting the disposition. Young patients with a history of anabolic steroid use should also be evaluated for tendinous disruption due to decreased tensile strength of the tendons secondary to steroid-induced dysplasia of the collagen fibrils.^11^

Musculoskeletal POCUS was performed with a high-frequency 5–10 megahertz linear probe throughout the distribution of the pectoralis muscle groups with special attention given to the distal portion of the pectoralis major muscle. Abduction and external rotation of the left arm were used to provide robust images of the distal pectoralis major under tension. Healthy pectoralis major muscles should appear hyperechoic and fibrillar throughout the clavicular and sternoclavicular heads with a uniform and taut tendon attaching to the bicipital groove of the humerus.^12^,^13^ In this patient, the muscle bellies of the pectoralis major were identified with obvious disruption of a distal muscle belly present. The observation of hypoechoic interruptions of muscular striation proximal to the musculotendinous junction, distal hematoma formation, and retraction of the distal pectoralis major muscle with an intact tendon suggested rupture of the distal pectoralis major muscle without major tendinous disruption.^14^,^15^

## CONCLUSION

Prompt evaluation, radiographic confirmation, and surgical intervention is key in ensuring positive outcomes in active patients with pectoralis major tears. Therefore, use of POCUS to aid in the diagnosis is pertinent when attempting to expedite disposition and improve patient care. In this case, POCUS evaluation was an effective adjunct in estimating the extent of injury and promptly involving orthopedic surgery to begin surgical planning with MRI.
